# Disability Bias in Patient Prioritization: How Patients’ Mobility Impairments Influences Prioritization to Mechanical Ventilation

**DOI:** 10.1007/s11606-026-10350-5

**Published:** 2026-04-28

**Authors:** Sagit Mor, Maya Peled-Raz, Adi Goldiner, Yafit Gafri, Michael Egozi, Ayelet Shai

**Affiliations:** 1 Faculty of Law, University of Haifa, Haifa, Israel; 2 International Center for Health Law and Ethics, University of Haifa, Haifa, Israel; 3 School of Public Health, University of Haifa, Haifa, Israel; 4 Cheshin Center for Advanced Legal Studies, Hebrew University of Jerusalem, Jerusalem, Israel; 5 Faculty of Medicine, Bar-Ilan University, Zafed, Israel; 6 Technion Israel Institute of Technology, The Ruth and Bruce Rappaport Faculty of Medicine, Efron st. 1, Haifa, Israel; 7 Oncology Wing, RAMBAM Heath Care Campus, Haifa, Israel; 8 Independent Researcher, Jerusalem, Israel

**Keywords:** access to healthcare, persons with disabilities, disabled patients, paralysis, mobility impairment, medical prioritization, medical triage, clinicians’ attitudes

## Abstract

**Background:**

During the COVID-19 pandemic, prioritization guidelines raised concerns that disabled patients would be de-prioritized for life-saving procedures, but empirical evidence is lacking.

**Objectives:**

To examine how physicians prioritize hypothetical patients with mobility impairments and to test the association between their attitudes toward disabled people and their prioritization preferences.

**Methods:**

Physicians and medical students completed a questionnaire comprising a vignette asking them to prioritize eight hypothetical patients needing mechanical ventilation. Each patient was described by a combination of paralysis from the waist down (yes/no), parenthood (yes/no), and employment (yes/no), reflective questions on the prioritization process, and attitudes towards people with disabilities and demographics. De-prioritization was defined as prioritizing a disabled patient lower than a similar patient without a disability. We tested the associations between attitudes towards people with disabilities, considerations taken during the prioritization process, and de-prioritization.

**Results:**

A total of 61.6% (77/125) of participants made de-prioritization decisions, with 38.4% de-prioritizing all hypothetical patients with disabilities. Negative attitudes toward patients with disabilities predicted de-prioritization (*b* = 0.75, SEb = 0.37, OR = 2.12, *p* = 0.041). Mediation analyses revealed an association between negative attitudes and considering patients’ presumed social and familial roles in the prioritization process, which was associated with de-prioritization of disabled patients (*b* = 0.26, SE = 0.16, 95% CI = 0.03,0.65, *p* < 0.001).

**Conclusion:**

This study indicates that during shortage in medical resources physicians may de-prioritize patients with lower limb paralysis without sound clinical justification. It suggests that negative attitudes towards people with disabilities predict employing non-medical considerations during prioritization, which are associated with discrimination against such patients in need of emergency interventions. We recommend enhancing physicians’ education about potential biases towards people with disabilities and implementing ethical guidelines to ensure fair prioritization of medical resources and equitable healthcare for these patients.

**Supplementary Information:**

The online version contains supplementary material available at https://doi.org/10.1007/s11606-026-10350-5.

## INTRODUCTION

Despite recognition of the right to equal healthcare for disabled people,^1–4^ they often receive inferior care that negatively impacts their health.^5–12^ While barriers stemming from features of the built environment and medical equipment^6,13,14^ and financial barriers^15^ can be addressed with legislation and funding, professional barriers such as communication barriers,^16,17^ stigmatizing or discriminatory attitudes,^16,18,19^ and professional standards and skills^11,19–21^ are harder to overcome. Studies showed that biased views of disabled people are prevalent among physicians.^18,22,23^ Ableism, which includes the perception of the able body and mind as essential for proper function and quality of life and the practices that favor abled persons,^24^ is still common in medical practice^25^ and education.^23,26,27^ Modern medicine is criticized for viewing disabilities through the lens of the biomedical model, and overlooking the social, cultural, and political constructs that shape disabled person’s lives and opportunities,^25,28,29^ and for failing to provide disability competent and affirming care.^12,29^

Prioritization of patients to scarce medical resources raises complex ethical debates.^30^ It is agreed that prioritization should rely on values of equity and fairness. Acceptable considerations include patients’ chances of survival and some advocated prioritizing people that risk their lives while performing professional duties.^31,32^ The impact of prioritization on disabled patients came to the forefront during the COVID-19 pandemic, when shortage of ventilators was expected.^31^ Many triage protocols placed disabled persons at the end of the queue and were fiercely criticized for devaluing disabled people’s lives (the USA;^33–35^ Canada;^36^ UK;^37^ Israel^38^). Ultimately, political and legal efforts promoted adoption of disability-neutral language;^39^ however, concerns that disabled patients would be de-prioritized persisted, due to the wide discretion of physicians in situations needing rapid responses.^39^

Prioritization of life-saving resources happens daily in healthcare systems with limited resources (i.e., prioritization of intensive care beds).^40^ Nevertheless, prioritization occurs sporadically, involves numerous factors, and is rarely documented, and empiric evaluation of prioritization and its association with patients’ disability raises considerable methodological difficulties. Brodar et al. reported that 2nd year medical students prioritized a woman with Down syndrome higher than abled patients during a prioritization exercise;^41^ however, in this exercise, the other fictional patients were older and much sicker. Empirical studies testing the prioritization of patients with disabilities and controlling for medical variables are however lacking. Against this backdrop, our aim was to explore whether prioritization of patients for scarce medical resources would disadvantage disabled patients. To overcome the difficulty of tracing undocumented prioritization decisions and to isolate the effect of disability on prioritization, we conducted an experimental study with a prioritization dilemma involving hypothetical patients with and without mobility disability. We hypothesized that, in the face of a shortage, patients with mobility disability will be deprioritized, even without solid clinical justification. We also hypothesized that decisions to de-prioritize disabled patients would be associated with underlying stigmatizing attitudes towards disabled persons.

## METHODS

### Participants

The study population included Israeli physicians working in hospital settings and medical students in their final year of clinical training.

### Sampling and Recruitment Methods

We recruited participants from September 2021 to June 2022 by sending a request to participate in a study on prioritizing medical resources through professional groups and directly to fellow medical professionals and medical students, and they forwarded the request to their colleagues. Those that agreed clicked on a link to an online questionnaire and completed it anonymously.

### Questionnaire

The questionnaire comprised four sections (see the translated questionnaire in Appendix 1):

- Vignette: Prioritization Dilemma

**Figure 1:**
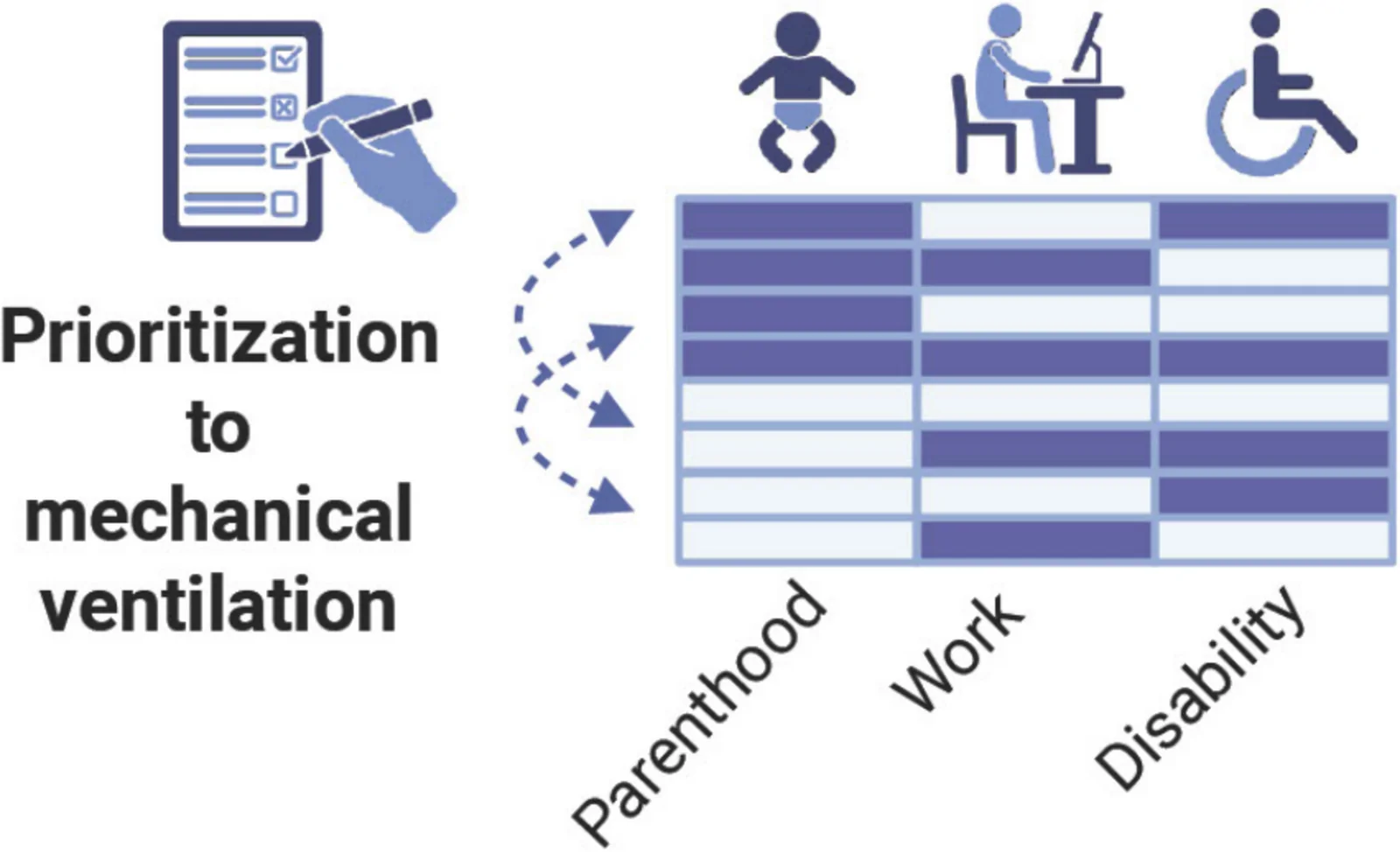
Schema of the experimental prioritization dilemma.

Participants were asked to decide the order in which they would allocate mechanical ventilators to eight hypothetical patients. All patients were 50-year-old males hospitalized for pneumonia complicated by respiratory failure. Each patient’s description included three additional variables: paralysis from the waist down from the age of 22 (yes/no), parent (yes/no), and employment status (yes/no) (Fig. 1). Lower limb paralysis was chosen to mitigate concerns about patient’s physical fitness, since it is well known that people with this disability can be physically fit like, or more than, abled people (e.g., athletes). Parenting and employment were introduced to mask the research question and to reduce the likelihood of social-desirability bias since these variables represent socially desirable traits rather than identity-based traits (e.g., gender, race, age). Participants were asked to rank patients for mechanical ventilators on a scale of 1 (highest) to 8 (lowest) and were allowed to assign multiple patients to each prioritization order (e.g., rank all patients 1 st).

(b) Considerations

Participants were asked to rank the extent by which different considerations influenced their decision-making process, on a Likert scale of 1–5 (1 = no influence, 5 = significant influence). These included the patient’s chances of dying in the ICU; the likelihood of the patient living without ventilator support; the patient’s contribution to society; the burden a patient places on his family; whether the patient is a parent; the patient’s quality of life; and the fact that the patient is paralyzed. These statements were chosen in accordance with previous literature on implicit and explicit disability bias.^18,42^

(c) Attitudes Towards People with Disabilities

Participants were asked to rate their agreement with statements describing general attitudes towards people with physical disabilities and attitudes concerning their medical care, on a Likert scale of 1–5, 1 = strong disagreement and 5 = strong agreement. This section was developed based on three previously validated questionnaires.^22,42,43^ After initial translation to Hebrew, the tools were back translated to English by an independent party to verify accuracy. Statements 12, 15, 17, 18, 20, and 21 were adapted to fit the context of the study (mobility disability instead of cognitive disability).

(d) Demographic Information

Participants provided their age, gender, years of professional experience, work location, specialty training, position, and native language. Participants also reported prior acquaintance and professional interactions with disabled people and receiving training on treating disabled patients.

### Validation

The questionnaire was peer-validated by a formal review of six social science experts for its relevance to the Israeli context. Subsequently, a pilot evaluation by five medical practitioners was undertaken, who completed the questionnaire and provided feedback regarding clarity and relevance to Israeli medical practice.

### Statistical Analysis

Disability-based de-prioritization was evaluated by comparing prioritization decisions made in four “pairs” of hypothetical patients, each pair comprised two patients with identical characteristics aside from the presence or absence of a disability. A decision was considered “de-prioritizing” when the disabled patient was prioritized lower than the non-disabled one. Decisions favoring disabled patients were given a negative score.

For each pair, a dichotomous variable was computed indicating whether the participant deprioritized disabled hypothetical patients. Each participant was denoted a dichotomous variable, reflecting whether they de-prioritized any of the disabled patients and the number of de-prioritizing decisions was calculated for each participant (0–4). An *F*-test was conducted to examine whether de-prioritization differed between the four patient pairs.

We next tested if 1) prioritization was associated with attitudes toward disabled people and towards their medical care. 2) Prioritization was associated with the considerations employed during prioritization.

An exploratory factor analysis (EFA) was conducted separately for attitudes toward disabled people (15 items), attitudes toward medical care of disabled patients (13 items), and the considerations employed during prioritization (seven items). The number of factors to retain was determined using parallel analysis. Items were considered to load onto a factor if they met the criterion of a minimum loading of 0.40 and were excluded if they loaded by less than 0.4 onto their factor and/or showed substantial cross-loading, defined as a difference of less than 0.20 between the items’ loading on the primary factor and its loading on any other factor.

Three binary logistic regression models were conducted, with the sub-scales questionnaires extracted in the EFA as independent variables and discrimination (no, yes) as the dependent variable.

Next, a sensitivity analysis was conducted, employing parallel linear regression analyses to test associations of the indices derived from the EFAs with a continuous measure of de-prioritization (0–4). Evaluation of all standard regression assumptions, including normality of residuals (via normal *P–P* plots), the presence of outliers (standardized residuals within ± 3 and Cook’s distance < 1), homoscedasticity, and multicollinearity (tolerance and VIF values) preceded these analyses. All assumptions were met, allowing reliable interpretation of the models.

Finally, we conducted two separate mediation models using Hayes’ PROCESS macro (Model 4: 5000 bootstrap samples, bias‑corrected 95% Cis^44^). The dependent variable was de‑prioritization (binary); the independent variables were the factors representing attitudes (the alternative attitude variable included as a covariate). The mediators were the factors representing considerations employed. Mediators were entered simultaneously, representing a parallel mediation model. All path coefficients (*a*, *b*, *c*′) were identical across both models due to statistical control. Significance of indirect effects was determined by examining the 95% bias-corrected bootstrap confidence intervals and was considered statistically significant if the confidence interval excluded 0.

Additional analyses examined whether background variables were associated with de-prioritization. Associations with categorical background variables were tested using chi-square tests or Fisher’s exact tests when assumptions were violated, and association with age was examined using an independent samples *t*-test. Effect sizes (Phi or Cohen’s *d*) were calculated where appropriate.

Missing data were handled using listwise deletion. Participants with incomplete responses on the variables required for a given analysis were excluded from that analysis (*n* = 7, 5.3%).

### Ethics

The study was approved by the Research Ethics Committee of University of Haifa (approval 255/21).

## RESULTS

### Participants

Out of 125 participants, 50% were female, about 67% native Hebrew speakers, and 17% native Arabic speakers. Table 1 details demographic and professional information of the 125 participants and Table 2 their prior acquaintance with persons with disabilities.

**Table 1:** Demographic Characteristics of Participants

		%	*N*
Gender	Female	50.4	63
	Male	49.6	62
Position	Department head	18.4	23
	Deputy department head	2.4	3
	Senior physician (consultant)	32.8	41
	Resident	14.4	18
	Intern	5.6	7
	Medical student (clinical rounds)	21.6	27
	Other	4.8	6
Specialty type	Medical/internal	44.83	52
	Surgical	25	29
	None reported	30.17	35
Native language	Hebrew	66.4	83
	Arabic	16.8	21
	Russian	8	10
	English	4.8	6
	Other	4	5

**Table 2:** Responses to Questions Regarding Prior Acquaintance with People with Disabilities

		%	*N*
I personally know a person with disabilities	Yes	81.6	102
	No	18.4	23
How often do you encounter patients with disabilities in your clinical work?	Never	5.04	6
	Once a month	24.37	27
	Once a week	36.97	44
	Once a day	12.61	15
	Several times each day	21.01	25
Did you have training on the treatment of people with disabilities?	Yes	6.4	8

### Prioritization and De-prioritization of Hypothetical Patients

**Figure 2:**
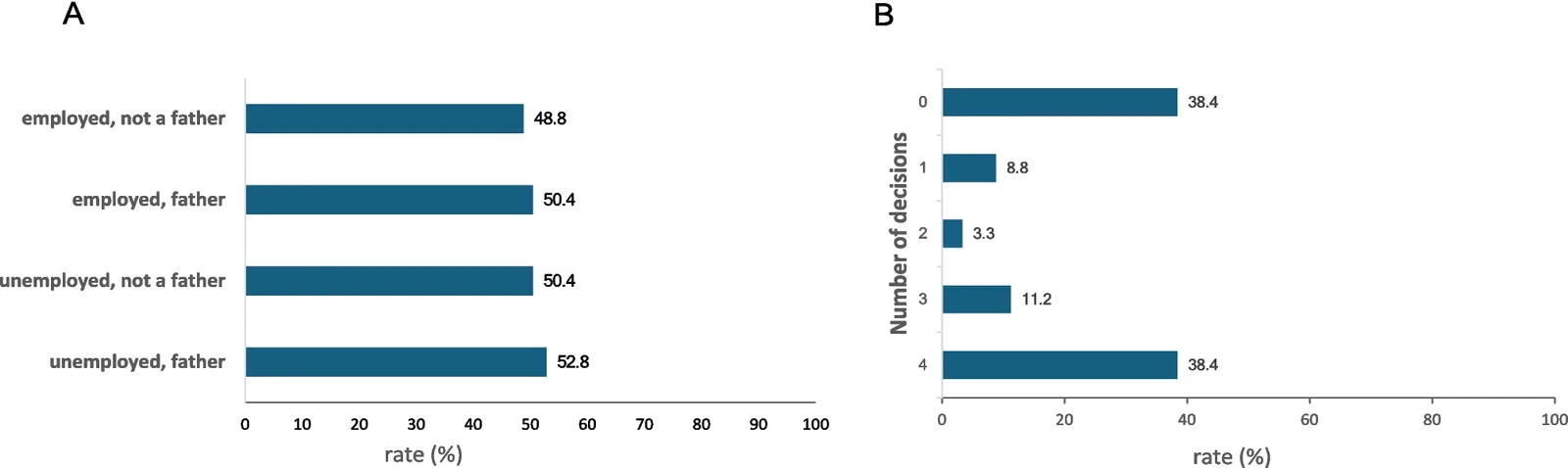
Rates of de-prioritization decisions. **A** De-prioritizing rates per patient pairs. **B** Ratio of participants per number of de-prioritization decisions.

Figure 2A illustrates the proportion of de-prioritization decisions across the four patient pairs. No significant differences in de-prioritization were detected between the pairs, *F*(3,122) = 0.71, *p* = 0.547.

Overall, 61.6% of participants de-prioritized a disabled patient in at least one pair (Fig. 2B) and 38.4% de-prioritized the disabled patients in all four pairs. Fewer participants de-prioritized disabled patients in one to two pairs.

### Association of Prioritization with Attitudes and Considerations

For details on factor analysis, see Appendix 2. Exploratory factor analysis of the statements on general attitudes toward people with disabilities resulted in two factors we defined as “negative attitudes” and “non-discriminatory attitudes.” Negative attitudes included viewing persons with disabilities as a burden on their families and society, viewing them as being dependent and having a lower quality of life, feeling uncomfortable around persons with dishabilles, thinking that society should not invest in promoting their rights, and that they would have been better off if they haven’t been born (Table 3). “Non-discriminatory attitudes” included viewing persons with disabilities as being able to live full and productive life, to have families and integrate in the labor market and to contribute to society, supporting state actions to promote rights of people with disabilities, and viewing them as equal to non-disabled people.

**Table 3:** Exploratory Factor Analysis of Attitudes Toward People with Disabilities

	Factor	
	1	2
**Non-discriminatory attitudes toward people with disabilities**		
Most people with disabilities can contribute to society through their experience and abilities	**0.84**	0.09
Most people with disabilities can work in the open labor market	**0.78**	0.00
Most people with disabilities are capable of having fulfilling romantic and family lives	**0.75**	− 0.05
Most people with disabilities can live full and satisfying lives	**0.68**	− 0.15
Support from state authorities can enable people with disabilities to live full and meaningful lives	**0.60**	0.19
People with disabilities are equal in value to people without disabilities	**0.51**	− 0.02
**Negative attitudes toward people with disabilities**		
Most people with disabilities are a burden on their family members	0.04	**0.82**
Most people with disabilities are a burden on society	0.03	**0.74**
The quality of life of most people with disabilities is low due to their disability	0.03	**0.62**
Investing in promoting the rights of people with disabilities is not worthwhile because their contribution to society is minimal	0.03	**0.51**
Being around people with disabilities usually makes me feel uncomfortable	0.13	**0.51**
Most people with disabilities cannot live independently	− 0.25	**0.49**
It would have been better if most people with disabilities had not been born	− 0.08	**0.48**
***Cronbach’s alpha***	**0.78**	**0.72**

Binary logistic regression was significant with the two attitude indices as independent variables and de-prioritization as the dependent variable (*XX* (2) = 17.42, *p* < 0.001, Nagelkerke* R*^2^ = 0.177). As expected, negative attitudes were associated with a higher likelihood of de-prioritization (*b* = 0.75, *SE*_*b*_ = 0.37, *OR* = 2.12, *p* = 0.041). Conversely, non-discriminatory attitudes were associated with a lower likelihood of de-prioritization (*b* = − 0.89, *SE*_*b*_ = 0.33, *OR* = 0.41, *p* = 0.007).

A sensitivity analysis by parallel linear regression using the continuous de-prioritization count yielded a similar pattern of associations and strengthens the robustness of the findings. The model was significant, *F*(2,122) = 7.12, *p* = 0.001, *R*^2^ = 0.104, with negative attitudes positively associated de-prioritization (β = 0.21, *p* = 0.021), and non-discriminatory attitudes negatively associated with de-prioritization (β = − 0.20, *p* = 0.028).

Exploratory factor analysis of the 13 items measuring attitudes toward medical care of patients with mobility disabilities resulted in two main factors we defined as “positive” and “negative.” Positive attitudes included welcoming patients with disabilities into one’s clinic and agreement that the state should provide disabled patients the care they need, and “negative” attitudes included viewing the care of disabled patients as pointless, tiring and wasting too much time and resources, and preferring not to treat patients with disabilities (Table 4).

**Table 4:** Exploratory Factor Analysis of Attitudes Toward Clinician–Patient Interaction with People with Disabilities

	Factor	
	1	2
**Negative attitudes toward providing care to people with disabilities**		
Treating people with disabilities requires too many resources	**0.71**	0.28
Treating people with disabilities takes too much time	**0.79**	0.21
Treating people with disabilities does not contribute to my professional advancement or prestige	**0.50**	− 0.26
Treating people with disabilities is pointless	**0.61**	− 0.27
Taking a medical history (anamnesis) from a patient with a disability is a tiring task	**0.58**	− 0.17
If I had a choice, I would prefer to see patients without disabilities rather than patients with disabilities	**0.59**	− 0.09
**Positive attitudes toward providing care for people with disabilities**		
The state should provide people with disabilities the medical care they need	− 0.12	**0.60**
I welcome patients with disabilities in my clinic/department	0.06	**0.86**
As a physician, it is very important to me to understand my patients who have a disability	0.04	**0.88**
***Cronbach’s alpha***	**0.69**	**0.75**

There was no significant association between attitudes towards medical care (either positive or negative) and de-prioritization, (chi-square value of (2) = 3.14, *p* = 0.208, and a Nagelkerke *R*^2^ of 0.034).

A sensitivity analysis by parallel linear regression using the continuous de-prioritization count was similarly non-significant *F* (2,122) = 0.90, *p* = 0.410, *R*^2^ = 0.015, and neither positive (*β* = 0.05, *p* = 0.623) nor negative (*β* = 0.12, *p* = 0.190) attitudes toward medical care of disabled patients were associated with de-prioritization.

Exploratory factor analysis of the seven items describing considerations employed during the prioritization process resulted in two factors we defined “considerations related to patient prognosis,” including chances of death and chances of being weaned off the ventilator and “considerations related to presumed social and familial roles,” including contribution to society, being a burden, being a father, and the fact that the patient is paralyzed (Table 5).

**Table 5:** Exploratory Factor Analysis of the Considerations Employed During Prioritization

	Factor	
	1	2
**Patient prognosis**		
Likelihood that the patient will die during ICU hospitalization	− 0.16	**0.95**
Likelihood that the patient will live without dependence on a ventilator	0.19	**0.88**
**Social and familial role**		
The extent to which the patient contributes to society	**0.95**	− 0.13
The extent to which the patient burdens their family	**0.85**	0.07
The fact that the patient is a father	**0.83**	− 0.04
The fact that the patient is paralyzed	**0.75**	0.13
***Cronbach’s alpha***	**0.87**	**0.81**

**Figure 3:**
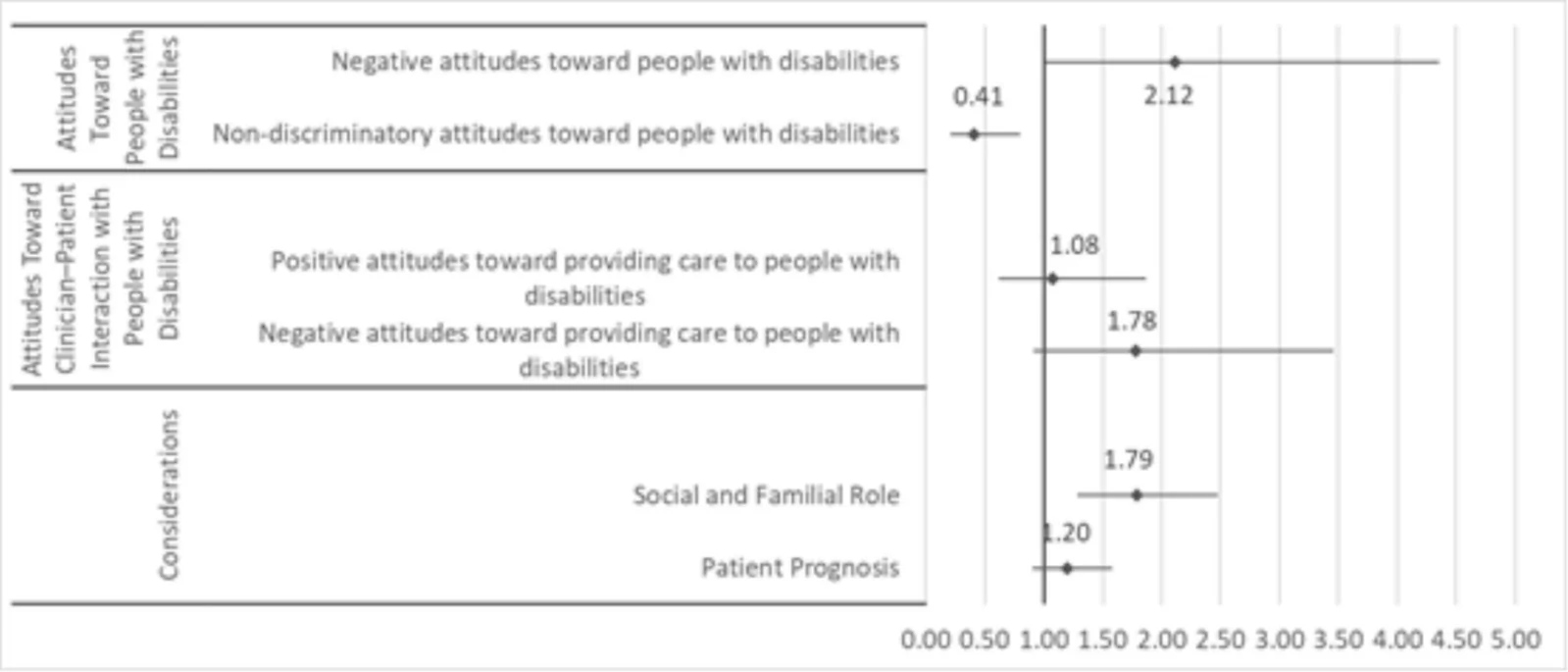
Forest plot of odds ratio and 95% CI for the association of attitudes and prioritization considerations with likelihood of de-prioritization. Values presented in the figure represent odds ratios (OR), with error bars indicating the 95% confidence intervals (CI) for OR.

Binary logistic regression analysis was significant, with two consideration indices as independent variables and de-prioritization as the dependent variable (*XX *(2) = 17.92, *p* < 0.001, Nagelkerke* R*^2^ = 0.181). Considering presumed social and family roles was associated with a higher likelihood of de-prioritization (*b* = 0.58, *SE*_*b*_ = 0.17, *OR* = 1.79, *p* < 0.001). In contrast, considering patients’ prognosis was not associated with de-prioritization (*b* = 0.18, *SE*_*b*_ = 0.14, *OR* = 1.20, *p* = 0.187). Figure 3 summarizes the associations between attitudes and considerations and likelihood of de-prioritizing patients with mobility disability.

A sensitivity analysis using parallel linear regression assessing the continuous de-prioritization count was likewise significant, *F* (2,122) = 5.97, *p* = 0.003, *R*^2^ = 0.089. Considering presumed social and familial roles was positively associated with the number of de-prioritization decisions (*β* = 0.26, *p* = 0.004), whereas prognosis-based consideration was not (*β* = 0.11, *p* = 0.200).

### Parallel Mediation of the Association Between Attitudes Toward Disabled People and De‑prioritization by Considerations

**Figure 4:**
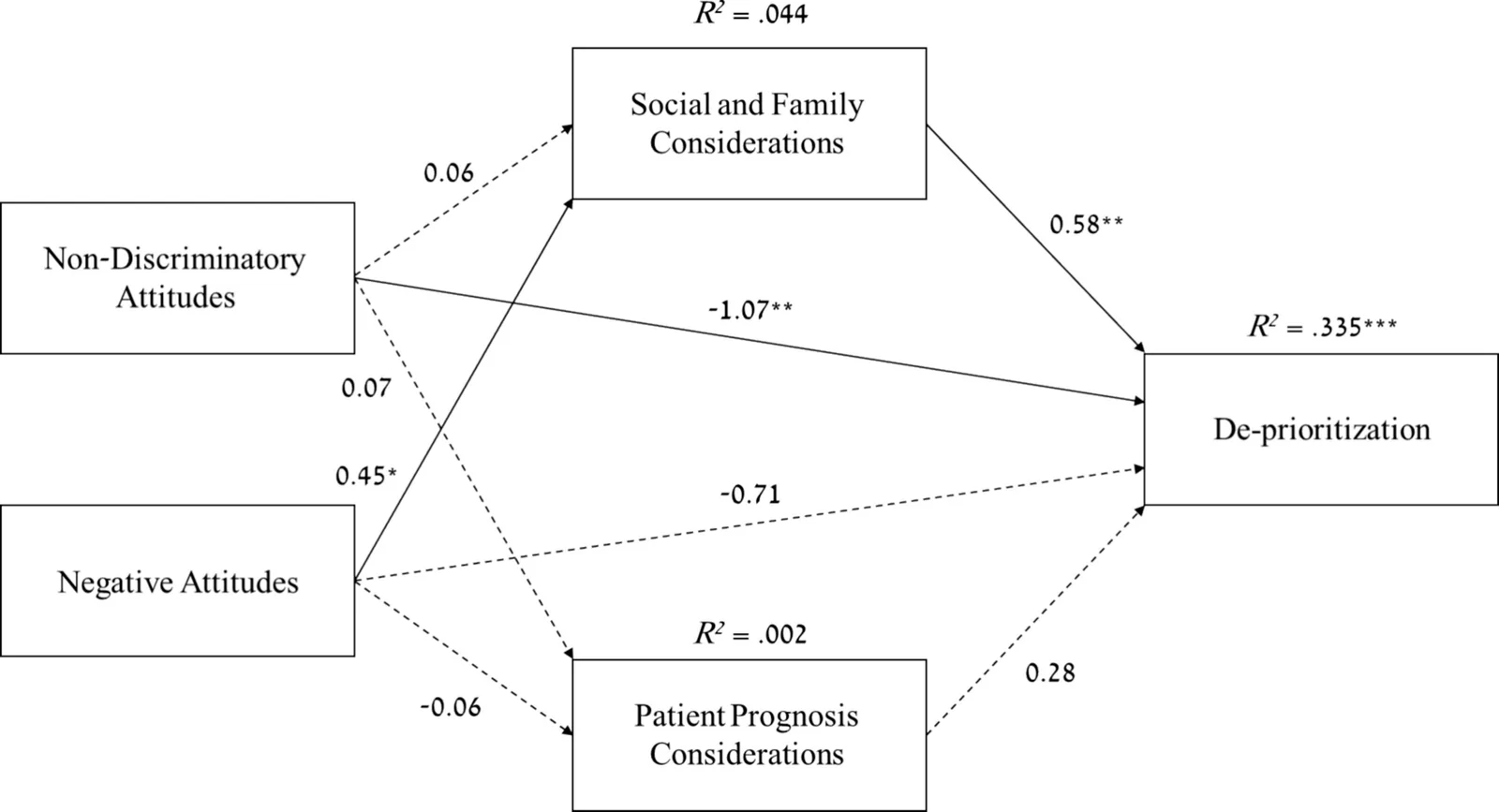
Parallel mediation of the association between attitudes toward people with mobility disability and de‑prioritization by the considerations employed during prioritization. *p < 0.05, **p < 0.01, ***p < 0.001.

Parallel mediation models indicated that negative attitudes toward people with disabilities were associated with considering patients presumed social and familial roles during the prioritization process, which was associated with a higher probability of de-prioritizing patients with a disability (*b* = 0.26, SE = 0.16, 95% CI = 0.03, 0.65). We did not find additional indirect effects in these analyses (Fig. 4).

### Association of Prioritization with Professional and Demographic Characteristics

We conducted additional analyses examining whether de-prioritization was associated with participants’ demographic and professional characteristics. Professional status (student vs physician) was significantly associated with de-prioritization, with physicians (67.1% de-prioritization rate) more likely than medical students (44.1%) to de-prioritize patients with mobility disabilities, *χ*^2^ (1, *N* = 119) = 5.35, *p* = 0.021, *Φ* = 0.21. No significant associations were found for gender, *χ*^2^ (1, *N* = 125) = 0.19, *p* = 0.661, *Φ* = 0.04, age, *t* (123) = − 0.18, *p* = 0.860, *d* = − 0.03, work setting, *χ*^2^ (1, *N* = 125) = 2.03, *p* = 0.154, *Φ* = 0.13, or participation in disability associated training, Fisher’s exact *p* = 0.709.

## DISCUSSION

Prioritizing patients for medical resources is rarely documented and difficult to study, and while concerns exist that disabled patients are de-prioritized for scarce medical resources,^4,33–35^ empirical evidence is lacking. 

Our study is the first to empirically examine, using an experimental design, how lower limb paralysis affects physicians’ preferences when prioritizing patients for emergency interventions while controlling for other variables. In this study, over 60% of participants prioritized disabled hypothetical patients lower than similar patients without disability, and viewing people with disabilities as dependent, being a burden on society and on their families, and having a lower quality of life was associated with de-prioritization. Moreover, we found that physicians with negative attitudes were more likely to consider the patient’s presumed familial and social roles during prioritization, and that these considerations were associated with de-prioritizing disabled patients.

While we cannot determine causality, these findings suggest that de-prioritization in our study resulted from biased views and negative attitudes towards people with mobility disabilities, and that during shortage in emergency resources, these biased attitudes influence professional judgment, resulting in detrimental effects for patients with disabilities.

Our findings also suggest that many physicians and medical students in Israel perceive persons with lower limb paralysis as less likely to contribute to society and as a burden, in line with studies of US physicians attitudes^18,20,43^ and training.^23,26^ Medical students were less likely to de-prioritize disabled patients in our study, possibly reflecting differences in moral standards and the effect of professionalism and burnout among physicians. Nevertheless, 44% of students in our study de-prioritized disabled patients without sound clinical reasoning. While most students in the exercise reported by Brodar et al.^41^ prioritized a hypothetical patient with Down syndrome higher than abled patients, this patient was younger and much healthier than abled hypothetical patients. Moreover, Brodar reported that some students prioritized patients according to social value,^41^ similar to our results. These findings may indicate that patients from other marginalized groups are at risk of under-treatment at times of resources shortage.

Disability scholars criticize assumptions about lower quality of life and inherent social futility of people with disabilities as stigmatizing and prejudiced. Studies found that, after an adjustment period, people with disabilities typically report quality of life levels similar to those of abled peers.^45,46^ Disability advocates argue that the assumptions that disability causes “suffering”,^47^ and that people with disabilities are a burden to society are incorrect and unjust, and that social structures generate economic dependence of people with disabilities.^48,49^

Our findings reveal a gap that must be filled in medical training, practice, and research. Medical education and healthcare institutions should address and remove potential biases against patients with disabilities. Prioritization practices that rely on perceived quality of life and contribution to society should also be addressed. Triage protocols should be continuously assessed and prognostic tools that mitigate disability bias should be developed. Better pandemic preparedness and mass triage planning with attention to patients with disabilities could reduce the risk of resource shortage and the detrimental effects of de-prioritization.

Our study has several limitations. A larger sample size might have helped us identify demographic and professional variables associated with prioritization. Since the invitation to participate in the study was circulated through social media, we were unable to assess response rates and characterize non-responders, and we cannot rule out sample bias. Randomization of the order of presentation was not performed, and we cannot rule out order effect. Of note, real-life decisions might be impacted by other clinical considerations and be subject to institutional ethical guidelines and interdisciplinary review. We did not test moral standards, religious faith, and additional value systems that may impact prioritization. Israel’s culture highly values family, parenting and reproduction,^50^ and our findings regarding the role of presumed familial roles in prioritization may not be applicable to cultures that differ from Israel in this regard. Finally, our study tested the impact of lower limb paralysis on prioritization to mechanical ventilation, and our results cannot be generalized to all types of disabilities and to other kinds of scarce medical resource allocation. The effect of specific disabilities on prioritization for mechanical ventilation and for additional medical interventions should be examined in further studies.

## CONCLUSION

This study indicates that, during shortage of medical resources, discriminatory prioritization decisions are likely to occur, disadvantaging patients with lower-limb paralysis, and that negative attitudes towards people with mobility disabilities are the drivers of these decisions. This suggests that the need to prioritize patients for scarce medical resources could generate barriers to equal access to healthcare for disabled people.

## Supplementary Information

Below is the link to the electronic supplementary material.

Supplementary file1 (DOCX 25.4 KB)

Supplementary file2 (DOCX 18.3 KB)

## Data Availability

Data will be available following a reasonable request from the corresponding author.
