# Supplementary material for: Disability Bias in Patient Prioritization: How Patients’ Mobility Impairments Influences Prioritization to Mechanical Ventilation
Source: J Gen Intern Med. 2026 Apr 28;41(12):3403–12. doi: 10.1007/s11606-026-10350-5 (PMC13569746; doi:10.1007/s11606-026-10350-5)
Supplement: Supplementary file 1 [file 11606_2026_10350_MOESM1_ESM.docx]

**Survey: Prioritizing the Provision of Medical Care**

In the following survey we ask you to respond to a series of questions concerning prioritizing the provision of medical care. The survey is divided into three parts. Each part contains the relevant instructions.

Part 1 – Case Descriptions

In the following you will be asked to make a decision regarding prioritization of medical care. Subsequently, you will be asked to rank the considerations that guided your choice.

1. The patients described below are hospitalized in the internal medicine department suffering from pneumonia. Their situation deteriorates and they require respiratory support.

**Please rank their priority on the waiting list for respiratory support** from 1 (first) to 8 (last). It is possible to rank several patients in to the same spot.

Patient A: Male, age 50, father of two, unemployed.

Patient B: Male, age 50, father of two, unemployed, paraplegic since the age of 22.

Patient C: Male, age 50, father of two, works at the municipal welfare department.

Patient D: Male, age 50, father of two, works at the municipal welfare department, paraplegic since the age of 22.

Patient E: Male, age 50, no children, unemployed.

Patient F: Male, age 50, no children, unemployed, paraplegic since the age of 22.

Patient G: Male, age 50, no children, works at the municipal welfare department.

Patient H: Male, age 50, no children, works at the municipal welfare department, paraplegic since the age of 22.

1. Please rank the considerations that guided your choice:

| Consideration | 1  Had no influence at all on my decision | 2 | 3 | 4 | 5  Significantly influenced my decision |
| --- | --- | --- | --- | --- | --- |
| a. Likelihood that the patient will die during hospitalization in the ICU |  |  |  |  |  |
| b. Likelihood that the patient will live without dependence on respiratory support. |  |  |  |  |  |
| c. Extent of the patient’s contribution to society |  |  |  |  |  |
| d. Extent of the burden that the patient places on his family |  |  |  |  |  |
| e. The fact that the patient is a father |  |  |  |  |  |
| f. The patient’s quality of life |  |  |  |  |  |
| g. The fact that the patient is paralyzed |  |  |  |  |  |
| h. I ranked them randomly |  |  |  |  |  |
|  | 1 Completely Disagree | 2 | 3 | 4 | 5  Largely agree |

2. Do you have any comments/anything you would like to add regarding the process of ranking and the considerations that guided you?

­­­­­­­­­­­­­­______________________________________________________________________________________________________________________________________________________________

Part 2 – Attitudes Survey

In this section you will be asked to indicate the degree by which you agree with a series of statements concerning people with physical disabilities and treating these people.

For the purposes of this survey, presume that “a person with disabilities” is a “person with a physical disability that significantly affects his functioning in one or more main areas of life.”

(Each page of the digital survey will contain the sentence: Please relate to physical disability).

B.1. Statements concerning general opinions regarding disabilities:

| Statement | 1  Completely disagree | 2 | 3 | 4 | 5  Largely agree |
| --- | --- | --- | --- | --- | --- |
| 1. Being in the presence of people with disabilities makes me uncomfortable |  |  |  |  |  |
| 2. People with disabilities are not treated equally in most areas of life |  |  |  |  |  |
| 3. People with disabilities are as valuable as those without disabilities |  |  |  |  |  |
| 4. Promoting the rights of people with disabilities is not worthwhile because they contribute little to society. |  |  |  |  |  |
| 5. Most people with disabilities are a burden on society |  |  |  |  |  |
| 6. Most people with disabilities are a burden on their families |  |  |  |  |  |
| 7. Most people with disabilities can contribute to society from their experience and their abilities |  |  |  |  |  |
| 8. Most people with disabilities can work in the free labor market |  |  |  |  |  |
| 9. Most people with disabilities have a lower quality of life due to their disabilities |  |  |  |  |  |
| 10. Most people with disabilities can lead a full and satisfying life |  |  |  |  |  |
| 11. It would be preferable for most people with disabilities that they had not been born |  |  |  |  |  |
| 12. Support from the state authorities will enable people with disabilities to life full and good lives |  |  |  |  |  |
| 13. Most people with disabilities cannot make decisions regarding their own lives |  |  |  |  |  |
| 14. Most people with disabilities cannot live independently |  |  |  |  |  |
| 15. Most people with disabilities can have fulfilling relationships and a fulfilling family life |  |  |  |  |  |

B.2. Statements concerning medical interactions and the healthcare system

| Statement | 1  Completely disagree | 2 | 3 | 4 | 5  Largely agree |
| --- | --- | --- | --- | --- | --- |
| 16. The state should provide people with disabilities with the medical care that they need |  |  |  |  |  |
| 17. Most people with disabilities are a burden on the healthcare system |  |  |  |  |  |
| 18. Healthcare professionals should receive specialized training to give the best possible medical care to people with disabilities |  |  |  |  |  |
| 19. With the right adjustments, caring for patients with disabilities is the same as treating those without disabilities |  |  |  |  |  |
| 20. Treating people with disabilities requires too many resources. |  |  |  |  |  |
| 21. The healthcare system is not suited to the needs of people with disabilities |  |  |  |  |  |
| 22. Treating people with disabilities takes too long |  |  |  |  |  |
| 23. Treating people with disabilities does not contribute to my professional advancement and prestige. |  |  |  |  |  |
| 24. Treating people with disabilities is pointless. |  |  |  |  |  |
| 25. Taking a medical history (anamnesis) from a patient with a disability is a tiring task |  |  |  |  |  |
| 26. If I had a choice, I would prefer to see patients without disabilities rather than patients with disabilities |  |  |  |  |  |
| 27. I welcome patients with disabilities in my clinic / department |  |  |  |  |  |
| 28. As a physician, it is important for me to understand my patients with disabilities |  |  |  |  |  |
| 29. |  |  |  |  |  |

Part 3 – Background Details

1. Age (years) ____
2. Gender: 1. Male	2. Female	3. Other – please provide details: ______
3. Position:
   a. Department head
   b. Deputy department head
   c. Senior physician
   d. Physician
   e. Intern
   f. Student - clinical stage
   g. Student – theoretical stage
   h. Other – please provide details _____________
4. If you are in the process of specialization / already a specialist, please note your area of specialization (if you are an intern or student, there is no need to answer this question)
   a. Internal medicine
   b. Emergency medicine
   c. Family medicine
   d. Surgery (including blood vessels, neurosurgery, ENT, urology, orthopedics, eyes, etc.)
   e. Gynecology
   f. Neurology
   g. Rehabilitation
   h. Oncology
   i. Hematology
   j. Pediatrics
   k. Geriatrics
   l. Other – please provide details: ________________
   m. Number of years working as a doctor (including process of specialization): ______________
5. I studied medicine in:
   a. Israel
   b. Eastern Europe
   c. Western Europe or the USA
   d. Other – please provide details ______________
6. Type of practice:
   a. Hospital.	1. General	2. Geriatric	3. Psychiatric (please indicate the type of hospital)
   b. HMO
   c. Independent clinic
   d. Combination: hospital and community medicine
   e. Other – please provide details _______________
   f. Not relevant (student)
7. Location of work: Southern Israel/ Central Israel / Northern Israel
8. Language spoken in parents’ home (circle): 1. Hebrew	2. Arabic	3. Russian	4. English	5. Amharic	6. Other – please provide details _________
9. Do you know someone with disabilities personally? 1. Yes	2. No
10. If so, in what framework did you make their acquaintance? (You can choose more than one answer)

|  | Type of disability | Framework of acquaintance |
| --- | --- | --- |
|  |  | I myself have a disability |
|  |  | Partner |
|  |  | Family member |
|  |  | Friend |
|  |  | Neighbor |
|  |  | Colleague |
|  |  | Other – please provide details_____________ |

11. How frequently do you encounter patients with disabilities in your work?
    a. Never	b. once a month	c. once a week	d. daily	e. several times a day
12. Have you participated in training concerning accessibility for people with disabilities?
    a. Yes	b. No

If yes, please indicate the name or focus of the training:_________________

In what framework was the training provided? ___________________________
