# Supplementary material for: Disability Bias in Patient Prioritization: How Patients’ Mobility Impairments Influences Prioritization to Mechanical Ventilation
Source: J Gen Intern Med. 2026 Apr 28;41(12):3403–12. doi: 10.1007/s11606-026-10350-5 (PMC13569746; doi:10.1007/s11606-026-10350-5)
Supplement: Supplementary file 2 [file 11606_2026_10350_MOESM2_ESM.docx]

**Factor analysis:**

***Attitudes toward people with mobility disabilities:***

In a primary analysis, exploratory factor analysis of the 15 items of attitudes toward people with mobility disabilities resulted in 2 factors we defined as “negative attitudes and “non-discriminatory attitudes”. Two items were removed due to cross-loading. See the 13 final items with their loadings and Cronbach's alpha in Table 3.

***Attitudes toward medical care of patients with mobility disabilities:***

In a primary analysis, exploratory factor analysis of the 13 items measuring attitudes toward medical care of patients with mobility disabilities resulted in 2 main factors we defined as “positive" and “negative” attitudes. Three items loaded on a third factor; however, they did not demonstrate coherent content and were excluded from the analysis. Additionally, one item was removed due to loading less than 0.4. The final analysis included nine items, along with their loadings and Cronbach's alpha, as seen in Table 4.

***Considerations taken during prioritization:***

In a primary analysis, exploratory factor analysis of the 7 items describing considerations employed during the prioritization process resulted in 2 factors we defined “considerations related to patient prognosis”, and “considerations related to presumed social and familial roles”. One item was removed due to content mismatch. See the 6 final items with their loadings and Cronbach's alpha in Table 5.
